# Use of a health information exchange system in the emergency care of children

**DOI:** 10.1186/1472-6947-11-78

**Published:** 2011-12-30

**Authors:** Joshua R Vest, 'Jon (Sean) Jasperson, Hongwei Zhao, Larry D Gamm, Robert L Ohsfeldt

**Affiliations:** 1 Health Policy & Management, Jiann-Ping Hsu College of Public Health, Georgia Southern University, 501 Forest Drive, Statesboro, GA, 30460, USA; 2 Department of Information & Operations Management, Mays Business School, Texas A&M University, 4217 TAMU, College Station, TX, 77843, USA; 3 Department of Epidemiology & Biostatistics, School of Rural Public Health, Texas A&M Health Science Center, 1266 TAMU, College Station, TX, 77843, USA; 4 Department of Health Policy & Management, School of Rural Public Health, Texas A&M Health Science Center, 1266 TAMU, College Station, TX, 77843, USA

## Abstract

**Background:**

Children may benefit greatly in terms of safety and care coordination from the information sharing promised by health information exchange (HIE). While information exchange capability is a required feature of the certified electronic health record, we known little regarding how this technology is used in general and for pediatric patients specifically.

**Methods:**

Using data from an operational HIE effort in central Texas, we examined the factors associated with actual system usage. The clinical and demographic characteristics of pediatric ED encounters (n = 179,445) were linked to the HIE system user logs. Based on the patterns of HIE system screens accessed by users, we classified each encounter as: no system usage, basic system usage, or novel system usage. Using crossed random effects logistic regression, we modeled the factors associated with basic and novel system usage.

**Results:**

Users accessed the system for 8.7% of encounters. Increasing patient comorbidity was associated with a 5% higher odds of basic usage and 15% higher odds for novel usage. The odds of basic system usage were lower in the face of time constraints and for patients who had not been to that location in the previous 12 months.

**Conclusions:**

HIE systems may be a source to fulfill users' information needs about complex patients. However, time constraints may be a barrier to usage. In addition, results suggest HIE is more likely to be useful to pediatric patients visiting ED repeatedly. This study helps fill an existing gap in the study of technological applications in the care of children and improves knowledge about how HIE systems are utilized.

## Background

Health information exchange (HIE), the process of electronically sharing identified, patient-level information between different organizations,[1] is a potentially transformative solution to problems of cost,[2] timeliness,[3] patient-centeredness,[4] safety,[3] and efficiency [5] that plague the healthcare system. Furthermore, children and adolescents may especially benefit from broad and easy information sharing. First, HIE has the ability to better support the care and detection of vaccine preventable conditions by incorporating immunization histories and linking to both local public health agencies and schools [4,6,7]. Second, minors constitute a substantial proportion of emergency department (ED) visits in the US,[8] with infants having the highest rates of ED visits [9]. The care delivered in the ED setting may benefit the most from improved information sharing [10,11]. Additionally, medication errors can be particularly dangerous for children; HIE improves communication and may prevent such mistakes [12,13]. Lastly, because HIE improves coordination among providers,[14] these information system can support providers in their provision of a medical home for all patients in general and children with special healthcare needs in particular.

Current federal policy dramatically advances the prospect for widespread HIE. The Health Information Technology for Economic & Clinical Health (HITECH) Act, part of the American Recovery & Reinvestment Act, identified information exchange capability and connectivity as a required feature of certified electronic health records (EHRs). To eligible for any EHR incentive payments, providers must now demonstrate Meaningful Use, which includes testing of HIE capabilities [15]. Despite the high level of support for HIE, we know very little about providers' motivations to use HIE systems or the effectiveness of HIE,[16-18] beyond the fact that these information systems are predominately accessed by a minority of providers [19] and for a minority of patients [20].

This paper aims to address the knowledge gap in HIE utilization regarding treatment of children. Previous researchers have argued for examinations of information technology for children separately from adult populations due to the particular vulnerabilities and unique needs of children [21,22]. In this examination, we address the question, what factors indicative of an information need or value of using HIE are associated with HIE usage? Both patient and encounter characteristics can change healthcare professionals' need for additional information. Factors such as patient complexity [23] or recent utilization [24,25] increase the uncertainty associated with delivering care and could prompt use of an HIE system [26]. Conversely, some encounters have little to do with the patient's previous utilization or are relatively uniformed by information created in other organizations. Likewise, the value of seeking potentially useful information may be lessened by other factors such as time constraints [27-29]. This study examines the factors associated with actual HIE usage during children's ED encounters.

## Methods

The Integrated Care Collaborative (ICC) of Central Texas is a fully functional HIE facilitating effort established in 1997 encompassing Austin, Texas area safety-net providers. The ICC exists as a separate nonprofit entity with 24 member organizations including: hospital systems, clinics, and governmental agencies. The study sample includes all ED encounters among patients less than 18 years old between 1/1/2006 and 6/30/2009 included in the ICC's master patient index/clinical data repository, I-Care. I-Care is a centralized database containing electronic patient level demographic and clinical information. ICC member organizations contribute patient level electronic data to I-Care on medically indigent patients. In turn, authorized users at each location may access data from I-Care through a secured website. Authorized users vary by location, but can include physicians, nurses, and/or administrative staff. Parents or guardians provide consent for minors to be included in the information exchange and this study only included consenting patients. We also excluded emergency encounters occurring at facilities before the hospital employed an authorized user of the I-Care system. The final dataset included 179,445 encounters from 11 emergency departments.

We derived the dependent variable representing type of usage from the I-Care system log files. Log files provide an objective and recommended [17,30] measure of system usage unbiased by subject recall [31]. The I-Care interface is an EpicWeb proprietary software system where authorized users navigate through several different web pages or screens containing demographics, prior utilization history, contact information, payer history, medication orders, prior diagnoses and other information. As part of the Health Insurance Portability and Accountability Act compliance, I-Care generates electronic log files in order to document the user's activities including: patient viewed, date accessed, time accessed, and screen(s) viewed. Through the logged date and time, we could follow the sequence of screens viewed by each user for a given patient on a given date. The entire sample included 77 different patterns of screen views in the associated log file. A single pattern accounted for 82% of sessions; this pattern consisted of an end user identifying a patient on a selection screen and then viewing a single screen containing a summary of recent encounters. We classified this type of session as *basic usage*. All other session patterns were classified as *novel usage*. A *novel usage session *consisted of any user session that included additional screen views (such as medications, a demographic summary, or detailed encounter records) beyond the initial patient selection screen and summary of recent encounters screen accessed in a basic usage scenario. A patient encounter in an ED could result in three usage outcomes: 1) no usage, 2) basic usage, and 3) novel usage. We linked user sessions to encounters based on patient identifier, date, user's work location, and place of encounter. Because ED encounters can occur late at night, we allowed for linkages up to 3 AM the next day.

We considered three factors as indicative of uncertainty that creates an information need: comorbidity, prior utilization, and unfamiliarity with the patient. The number of unique disease categories for each encounter measured comorbidity. Disease categories were defined by the Agency for Healthcare Research & Quality's (AHRQ) Clinical Classifications Software applied to all reported ICD-9 diagnosis codes [32]. For prior utilization, we determined the total number of ED encounters, inpatient hospitalizations, and primary care clinic visits at ICC member facilities in the 12 months prior to the encounter date. We did not include previous visits to the same ED in these counts. Following existing definitions of encounter frequency, we divided ED and primary care visits into 0 encounters, infrequent users (1 to 3), and frequent users (4 or more) [33-35]. Due to small cell counts, we could only consider hospitalization in the previous 12 months in a binary fashion. Finally, patients unfamiliar to a specific ED were marked by the absence of any encounters at that same ED in the previous 12 months. Because we excluded visits at the same facility from the measure of past ED encounters, we avoided collinearity for this measure of patient unfamiliarity.

To measure potential time constraints, we created a binary variable to classify the encounter date at that ED as busy or not busy. For each ED, we divided the total number of encounters on a date by the ED's previous year's average number of daily encounters for that same day of the week and month. A busier than average day existed when this ratio was greater than one.

We categorized the primary diagnosis and payer to help describe the sample. First, AHRQ's Chronic Condition Indicator and Body Systems definitions categorized the primary diagnosis as a chronic condition and assigned the primary diagnosis into 18 indicators roughly analogous to major diagnostic categories [36]. We selected factors influencing health status and all categories that occurred in less than 1% of encounters as the reference category for the analysis. We grouped the payer associated with the encounter into Medicaid, Children's Health Insurance Program (SCHIP), charity care (sliding scale, self-pay, or charity care) and multiple or no payers reported.

Hypotheses were examined using crossed random effects logistic regression models [37]. The random intercepts account for the clustering of encounters within patients and patients within EDs. The crossed effects allow for patients with encounters at different EDs. Since the reference category is substantially larger than the outcomes of interest, independent mixed effects binary logistic equations were fit instead of a multinomial logistic regression [38]. All variables are considered as fixed effects and the models were fit using Laplacian approximation [37]. Measures of effect size were expressed odds ratios (OR). The advantage of a random effect logistic regression model is its ability to model the correlated nature of the binary data arising from a multi-level structure; it is difficult to do so using the alternative approach of the generalized estimation equations. However, the interpretation of the fixed effects from the random effect logistic regression is not very transparent, since they carry a subject-specific meaning, instead of indicating a population averaged effect. In general, the population averaged effect is smaller than the subject-specific effect [39]. Hence, we need to keep this in mind when interpreting our regression models. In the special case with logit link and one random effect, Heagerty and Zeger [40] showed that the marginal parameters were reduced by a factor that depends on the variance of the random effect. Nonetheless, the significance levels of the parameter estimates from these two types of models often stay the same.

The project was approved the Institutional Review Boards of Georgia Southern University and Texas A&M University.

## Results

The system was accessed for 15,586 of 179,445 encounters (8.7%), which was higher than other published reports [2,20]. Table 1 describes the study sample. Most encounters were among males, Hispanics, and paid for by Medicaid. Patients aged 1 to 5 years old accounted for more than 4 out of 10 encounters. The four most common primary diagnoses involved ill-defined conditions (23.1%), diseases of the respiratory system (22.9%), injuries and poisoning (15.5%) and diseases of the central nervous system (10.7%).

**Table 1:** Characteristics of encounters at emergency departments among children included in the Integrated Care Collaboration, 1/1/2006-6/30/2009.

	All encounters n = 179,445	Encounters without HIE usage n = 163,859	Encounters with basic usage n = 12,910	Encounters with novel usage n = 2,676
Female, %	47.9	48.1	46.2	46.6

Age, %				

< 1 year	21.6	21.8	19.1	23.5

1 to 5 years	44.3	44.5	53.7	51.6

6 to 11 years	18.9	18.9	19.0	15.7

12 to 17 years	15.2	15.8	8.2	9.2

Race/ethnicity, %				

White non-Hispanic	19.1	19.6	13.5	13.0

African American	12.8	12.7	13.2	13.5

Hispanic	60.5	60.0	69.8	69.4

Other/unknown	8.0	3.4	4.0	7.6

Payer, %				

Medicaid	66.9	66.7	69.8	69.1

CHIP	5.3	5.3	4.5	2.8

Charity care	24.2	24.5	20.6	21.9

Unknown/multiple recorded	3.7	3.5	5.1	6.1

Utilization history*				

Clinic visits, %				

0	78.4	79.4	69.3	59.4

1 to 3	12.1	116.	16.5	20.2

≥ 4	9.5	9.0	14.2	20.4

Emergency department visits, %				

0	78.2	78.8	70.3	78.8

1 to 3	19.6	19.1	26.0	19.0

≥ 4	2.3	2.1	3.8	2.2

Hospitalized, %	14.2	13.9	17.0	20.4

Charlson index, %				

0	81.5	82.1	76.2	72.5

1	17.0	16.5	21.6	24.4

≥ 2	1.5	1.4	2.2	3.1

Chronic condition indicator, %	4.6	4.5	5.7	6.1

Primary diagnosis^†^, %				

Infectious & parasitic disease	5.7	5.7	5.9	6.5

Neoplasms	0.0	0.0	0.0	0.0

Endocrine, metabolic & immunity	0.5	0.5	0.6	0.5

Diseases of the blood	0.3	0.3	0.4	0.4

Mental disorders	0.3	0.3	0.1	0.3

Diseases of the nervous system	10.7	10.6	11.8	11.0

Diseases of the circulatory system	0.1	0.1	0.0	0.1

Diseases of the respiratory system	22.9	22.9	23.8	23.7

Diseases of the digestive system	4.4	4.3	4.4	5.2

Diseases of the genitourinary system	2.7	2.7	2.7	3.1

Complications of pregnancy/childbirth	0.7	0.8	0.1	0.5

Diseases of the skin	5.2	5.1	5.7	4.4

Diseases of the musculoskeletal	1.6	1.6	1.6	1.4

Congenital anomalies	0.1	0.1	0.0	0.0

Conditions originating in the perinatal period	0.4	0.4	0.1	0.0

Injury & poisoning	15.5	15.8	13.9	9.1

Ill-defined conditions	23.1	23.0	23.2	27.2

Factors influencing health status	5.9	5.9	5.9	6.8

Unfamiliar patient^‡^, %	52.5	54.4	33.9	16.8

Busier than average day^§^, %	59.0	60.2	44.4	56.5

* Not seen at the same facility in the past 12 months

^†^Agency for Healthcare Research & Quality's body system indicator categories

^‡ ^Not seen at the same facility in the past 12 months

^§^Ratio of the total number of same day encounters divided by the previous year's average number of daily encounters by month and day of week greater than 1.0

### Factors associated with basic usage

Table 2 describes the factors associated with basic usage unadjusted and adjusted for confounding. After controlling for confounding factors, several factors indicative of patient complexity increased the odds of basic usage. In terms of patient comorbidity, the odds of basic usage were 5% higher (OR = 1.05; 95%Confidence interval (CI) = 1.02, 1.08) for each additional recorded diagnosis category during the encounter. Specific to recent utilization history, increasing number of primary care visits, visits to other EDs, and prior hospitalization in the previous 12 months each increased the odds of basic usage. Contrary to expectations, the odds of basic usage were lower for unfamiliar patients (OR = 0.46; 95%CI = 0.44, 0.48).

**Table 2:** Association between patient, encounter, ED characteristics and basic health information exchange usage.

	Basic usage	
	**Unadjusted**	**Adjusted**

	OR (95%CI)*	**OR (95%CI)**

Female	0.97 (0.93, 1.01)	0.98 (0.94, 1.02)

Age (years)		

1 to 5 vs < 1	1.56 (1.48, 1.64)	1.68 (1.58, 1.79)

6 to 11 vs < 1	1.37 (1.29, 1.46)	1.78 (1.65, 1.91)

12 to 17 vs < 1	1.21 (1.12, 1.31)	1.54 (1.41, 1.69)

Race/ethnicity		

African American vs White^†^	1.09 (1.01, 1.18)	1.00 (0.93, 1.08)

Hispanic vs White^†^	1.03 (0.97, 1.10)	0.98 (0.93, 1.04)

Other/unknown vs White^†^	0.69 (0.61, 0.77)	0.72 (0.64, 0.81)

Payer		

SCHIP vs Medicaid	0.79 (0.72, 0.86)	0.93 (0.85, 1.03)

Charity care vs Medicaid	0.85 (0.83, 0.92)	0.96 (0.92, 1.01)

Unknown/multiple vs Medicaid	1.57 (1.43, 1.72)	1.48 (1.35, 1.63)

No. of primary care clinic visits^‡^		

1 to 3 vs 0	1.49 (1.41, 1.57)	1.47 (1.39, 1.55)

≥ 4 vs 0	1.58 (1.48, 1.67)	1.35 (1.27, 1.44)

No. of emergency depart visits to other facilities^‡^		

1 to 3 vs 0	1.75 (1.68, 1.84)	1.60 (1.52, 1.67)

≥ 4 vs 0	2.54 (2.28, 2.84)	2.19 (1.96, 2.45)

Hospitalized^‡^	1.12 (1.06, 1.18)	1.33 (1.25, 1.41)

No. of unique diagnosis categories	1.07 (1.05, 1.10)	1.05 (1.02, 1.08)

Chronic condition indicator	1.17 (1.08, 1.28)	1.10 (1.01, 1.20)

Primary diagnosis		

Infectious & parasitic disease	1.00 (0.90, 1.11)	1.00 (0.90, 1.11)

Diseases of the nervous system	1.16 (1.06, 1.27)	1.07 (0.97, 1.17)

Diseases of the respiratory system	1.06 (0.98, 1.27)	1.01 (0.93, 1.10)

Diseases of the digestive system	0.95 (0.85, 1.06)	0.99 (0.88, 1.11)

Diseases of the genitourinary system	1.13 (0.99, 1.29)	1.11 (0.97, 1.27)

Diseases of the skin	1.21 (1.09, 1.35)	1.19 (1.07, 1.52)

Diseases of the musculoskeletal	1.22 (1.03, 1.44)	1.28 (1.08, 1.52)

Injury & poisoning	1.06 (0.97, 1.16)	1.15 (1.06, 1.26)

Ill-defined conditions	1.02 (0.94, 1.10)	1.01 (0.93, 1.09)

All other categories^§^	1.00	1.00

Unfamiliar patient^||^	0.47 (0.45, 0.48)	0.46 (0.44, 0.48)

Busier than average day^¶^	0.64 (0.62, 0.67)	0.65 (0.62, 0.67)

Random part		

Variance (location)		15.84 (2.93, 85.65)

Variance (patient)		0.28 (0.24, 0.32)

* Odds ratio & 95% confidence interval

^† ^non-Hispanic

^‡ ^In past twelve months

^§ ^Including: neoplasms; endocrine, metabolic & immunity; diseases of the blood; mental disorders; diseases of the circulatory system; factors influencing health status

^|| ^Not seen at the same location in the past year

^¶^Ratio of total number of same day encounters divided by the previous year's average number of daily encounters by month and day of week

Time constraints also appear to be a barrier to usage. Odds of usage were 35% lower on busier than average days in the ED (OR = 0.65; 95%CI = 0.62, 0.67). In terms of primary diagnoses, the odds of basic usage were 19% higher for diseases of the skin, 28% higher for diseases of the musculoskeletal system and 15% higher for injuries and poisoning. In addition, encounters with a chronic condition had 10% higher odds of basic usage. Lastly, the odds of basic usage were higher for encounters with older pediatric patients and when more than one payer existed or the payer was unknown. The use of a random effects model is supported by the statistically significant variances for both location and patient.

### Factors associated with novel usage

Table 3 displays the factors associated with novel usage. After adjusting for other factors, patient comorbidity, measured as the number of diagnoses at the encounter, positively increased the odds of novel usage (OR = 1.15; 95%CI = 1.09, 1.21). In addition, prior utilization of primary care and hospitalization in the previous 12 months were both positively associated with novel usage. As was the case with basic usage, the odds of novel usage were lower for unfamiliar patients (OR = 0.19; 95%CI = 0.17, 0.21).

**Table 3:** Association between patient, encounter, ED characteristics and novel health information exchange usage.

	Novel usage	
	**Unadjusted**	**Adjusted**

	OR (95%CI)*	**OR (95%CI)**

Female	0.98 (0.90, 1.07)	0.97 (0.89, 1.06)

Age (years)		

1 to 5 vs < 1	1.17 (1.06, 1.30)	1.34 (1.19, 1.51)

6 to 11 vs < 1	0.87 (0.76, 1.00)	1.37 (1.17, 1.60)

12 to 17 vs < 1	0.91 (0.78, 1.08)	1.39 (1.15, 1.68)

Race/ethnicity		

African American vs White^†^	1.16 (0.98, 1.36)	1.00 (0.85, 1.18)

Hispanic vs White^†^	1.09 (0.98, 1.10)	0.82 (0.72, 0.93)

Other/unknown vs White^†^	0.69 (0.61, 0.77)	0.89 (0.70, 1.13)

Payer		

SCHIP vs Medicaid	0.49 (0.39, 0.62)	0.60 (0.47, 0.76)

Charity care vs Medicaid	0.92 (0.84, 1.02)	1.17 (1.06, 1.30)

Unknown/multiple vs Medicaid	1.75 (1.47, 2.09)	1.59 (1.32, 1.91)

No. of primary care clinic visits^‡^		

1 to 3 vs 0	2.20 (1.97, 2.45)	2.19 (1.96, 2.45)

≥ 4 vs 0	2.84 (2.54, 3.18)	2.24 (1.99, 2.52)

No. of emergency depart visits to other facilities^‡^		

1 to 3 vs 0	1.11 (1.00, 1.24)	1.04 (0.93, 1.16)

≥ 4 vs 0	1.22 (0.92, 1.62)	1.02 (0.76, 1.36)

Hospitalized^‡^	1.46 (1.31, 1.62)	1.39 (1.22, 1.57)

No. of unique diagnosis categories	1.22 (1.67, 1.29)	1.15 (1.09, 1.21)

Chronic condition indicator	1.22 (1.03, 1.44)	1.17 (0.97, 1.40)

Primary diagnosis		

Infectious & parasitic disease	1.00 (0.81, 1.23)	0.98 (0.79, 1.22)

Diseases of the nervous system	0.97 (0.81, 1.17)	0.88 (0.72, 1.06)

Diseases of the respiratory system	0.95 (0.81, 1.11)	0.88 (0.74, 1.04)

Diseases of the digestive system	1.00 (0.80, 1.25)	1.06 (0.84, 1.33)

Diseases of the genitourinary system	1.15 (0.88, 1.49)	1.15 (0.87, 1.50)

Diseases of the skin	0.81 (0.65, 1.03)	0.87 (0.68, 1.10)

Diseases of the musculoskeletal	0.93 (0.64, 1.34)	1.17 (0.81, 1.71)

Injury & poisoning	0.60 (0.50, 0.73)	0.80 (0.66, 0.97)

Ill-defined conditions	1.06 (0.91, 1.25)	1.05 (0.89, 1.23)

All other categories^§^	1.00	

Unfamiliar patient^||^	0.17 (0.16, 0.19)	0.19 (0.17, 0.21)

Busier than average day^¶^	1.04 (0.96, 1.12)	0.96 (0.88, 1.04)

Random part		

Variance (location)		25.87 (2.39, 280.11)

Variance (patient)		1.05 (0.90, 1.23)

* Odds ratio & 95% confidence interval

^† ^non-Hispanic

^‡ ^In past twelve months

^§ ^Including: neoplasms; endocrine, metabolic & immunity; diseases of the blood; mental disorders; diseases of the circulatory system; factors influencing health status

^|| ^Not seen at the same location in the past year

^¶^Ratio of total number of same day encounters divided by the previous year's average number of daily encounters by month and day of week

Controlled for other factors, a busier than average day at the ED was not with associated novel usage. Increasing age was associated with novel usage. After adjustment, the only diagnosis category associated with novel usage was injury and poisoning (OR = 0.80; 95%CI = 0.66, 0.97). Encounters where the payer was not Medicaid were associated with novel usage. The odds of novel usage were 40% lower for SCHIP and 17% higher for charity care. Again, the statistically significant variances indicated the appropriateness of the random effects model.

Novel usage and basic usage varied in several key respects. As noted above, encounters with a primary diagnosis of injury and poisoning had opposite associations with basic and novel usage. Second, utilization of other EDs increased the odds of basic usage, but was unassociated with novel usage. The same was true for encounters due to chronic conditions. Finally, how busy the ED was the day of encounter had different effects on the odds of usage: the odds of basic usage were lower, and no affect was seen on novel usage.

## Discussion

HITECH and the subsequent Meaningful Use criteria means HIE activity will become more widespread in the near future. However, healthcare professionals must actually use the information systems that make the information from HIE activities available before any benefits can accrue. This study provides insights into some of the general reasons for, and barriers to, HIE usage for pediatric emergency encounters. First, as anticipated, factors suggestive of more complex patients were associated with both basic and novel usage. Specifically, the more conditions present at the encounter and recent hospitalizations increased the odds of usage. Both of these factors are consistent with HIE usage research in adult populations,[26,41,42] and complex situations, in general, are a driver of information seeking [43]. Likewise, frequent primary care usage increased the odds of both basic and novel HIE usage. Patients with numerous encounters in other settings create interdependencies in the provision of care and increased information about those encounters could prove useful in patient care. However, the frequent use of other services may also be an indicator of ill health and therefore complexity.

Another traditional driver of information seeking, unfamiliarity, actually turned out to be negatively associated with both types of HIE usage. The unfamiliar patient is broadly assumed to justify information exchange,[10,25,44] and not without reason, as the unfamiliar patient results in a knowledge deficit potentially filled by HIE [18]. However, the odds of basic usage were lower for encounters where the patient not had been seen at the facility for at least year. We saw similar results in our previous study of HIE system usage among adult ED encounters [41] and recently Johnson and colleagues [45] also noted higher rates of HIE system access for patients' return visits to the ED. The odds of novel usage were even lower. It would appear that HIE usage, at least in the ED setting, is more likely to be useful for repeat patients. Possibly, for unfamiliar patients, more attention may be paid to obtaining a thorough history, reducing any perceived need for HIE or repeat visits may prompt providers to be more attentive to treatments and care from other locations. These counterintuitive findings underscore the need for future qualitative research to completely understand users' intentions in such settings and to identify steps to insure optimal use of available HIE information.

While this study does not measure the exact type of information sought, through the use of basic and novel usage classifications allows us to infer some ideas of information value. As would be expected with a voluntary use system, the busier the day the less likely the basic usage of the system. That is, when things got busy, users had lower odds of looking up basic, readily displayed summary information. This was also true in our study of adult populations [41]. In contrast, the opportunity costs incurred on a busy day did not negatively affect novel usage. This may suggest that conditions observed in the child may drive novel usage regardless of how busy the ED is, while basic usage is more easily suppressed. Information that was beyond the more in-depth or the not commonly reported, appeared worthwhile to seek out even during a busy day. In addition, charity care was associated with novel usage and encounters with unknown payers were positively associated with all types of usage. Usage during these encounters that could prove more costly to the facility may represent an attempt to locate more information about the patient or a history of payer eligibility. Finally, although previous research indicates a demand for lots of clinical data elements from HIE systems in the ED setting,[46] few diagnosis categories were associated with either type of usage.

The only primary diagnoses category associated with both types of usage was injury and poisoning, although importantly the direction of the effect differed. In general, one could assume injuries are a type of encounter where the diagnosis and treatment should not depend upon data stored in other organizations' information systems. While that view explains the negative association with novel usage it does not explain the positive relationship with basic usage. When specifically considering the case of children, other factors come into play that may explain this apparent discrepancy. Nationwide, a significant percentage of ED encounters among children are due to abuse [47]. The positive association with basic usage may be indicative of a quick check to determine if a history or pattern of injury may be discernible. This type of usage would be a great strength of HIE systems as it would detect perpetrators who utilize multiple EDs to hide repeat injuries to the same child or even vulnerable adults like the elderly or those with an intellectual disability. The positive association with novel usage is in contrast to our previous findings of HIE usage among adults ED encounters. Among adults, injuries were negatively associated with both types of usage,[41] which is logical as injuries to most adults do not carry a concern about abuse. We encourage future researchers to examine this relationship between ED injury encounters and HIE usage.

Overall, these results complement to the broader literature on the determinants of information seeking among clinicians. For example, research by Gorman and Helfand,[48] although focused on knowledge-based resources, reported information seeking was associated with the urgency of the patient's problem. A similar phenomenon was evident in this study, as an increasing number of diagnoses was associated with an increased odds of both types of system usage. Also focused on the use of knowledge-based resources, Ely and colleagues [49] identified a lack of time as a barrier; in like manner, we reported if the day had more encounters than average, the odds of routine usage decreased significantly. Lastly, investigators have begun to catalog the types of information desired by providers from HIE systems [46,50]. While our study does not look at system access for specific data elements, our measure of basic usage represents the factors associated with access of summary patient information.

### Limitations

The results of this study are limited in more than one fashion. First, this study does not include any direct measures of the system users. For any given encounter, the system examined in this study only records use. While we can identify encounters without usage, we have no way of attributing this non-use to an individual user. In other words, while we can identify the users who employed the system, we cannot identify the users who did not use the system. Therefore, we cannot address any potential confounding due to user characteristics like job type, computer skills, or perceptions of system usability. Likewise, we did not systematically explore the potential the role of workplace characteristics. For example, computer terminals may not have been equally accessible to users in each ED or the speed of internet connection may have varied. While our random-intercept models statistically adjust for these differences, we do not explicitly model their effects. Third, our measure of usage does not include the specific data sought, search strategies, encounter workflow, or if the search was successful. Previous research demonstrates end users need a wide variety of patient information types,[51] engage in the system in very diverse ways,[52] and tighter integration into workflow improves usage rates [20,53]. Despite these limitations our measure of usage is more informative than previous research [54]. The logical next step in research is to examine these suggested factors qualitatively with users and the context workflow. Fourth, due to the user location and date restrictions in the linking process, we have excluded users accessing the system after a patient encounter to identify patients for disease management programs, social services, or public health. The factors associated with usage could reasonably be expected to differ among these types of users. Finally, caution is needed in generalizing this study to other types of healthcare encounters or other HIE efforts since this particular exchange only includes medically indigent patients and one particular HIE system. Other HIE systems may differ on key characteristics such as breadth of information types and sources, the display of information, or overall system usability.

## Conclusions

These findings help fill a gap in knowledge about what type of HIE use occurs for children and under what conditions. Healthcare professionals, advocates and the government believe HIE has the potential to transform healthcare and children may benefit greatly from the improved information sharing. However, the simple existence of these systems is not likely to be sufficient and assuming use will automatically follow existence is unfounded. HIE systems must be applied to the delivery of care and the improvement of patient health. The improved use of HIE to avoid duplication and improve coordination of care for pediatric patients will be increasingly important as health reform moves to extend coverage to nearly all children.

## Competing interests

The authors declare that they have no competing interests.

## Authors' contributions

JV conceived the research question, designed the study, analyzed the data, and drafted the manuscript. 'JJ designed the dependent variables and drafted the manuscript. HZ led the statistical analysis. LG helped design the study and interpreted the results. RO helped design of the study, interpreted the results and draft the manuscript. All authors read and approved the final manuscript.

## Pre-publication history

The pre-publication history for this paper can be accessed here:

http://www.biomedcentral.com/1472-6947/11/78/prepub
